# Editorial: Translational imaging in neurodegenerative proteinopathies

**DOI:** 10.3389/fnins.2022.952449

**Published:** 2022-07-11

**Authors:** Ruiqing Ni, Naruhiko Sahara, Bin Ji

**Affiliations:** ^1^Institute for Regenerative Medicine, University of Zurich, Zurich, Switzerland; ^2^Institute for Biomedical Engineering, ETH & University of Zurich, Zurich, Switzerland; ^3^National Institute of Radiological Sciences, National Institutes for Quantum and Radiological Science and Technology, Chiba, Japan; ^4^School of Pharmacy, Fudan University, Shanghai, China

**Keywords:** proteinopathy, astrocytosis, PET, MRI, Alzheimer's disease, Parkinson's disease, SV2A

Neurodegenerative diseases, including Alzheimer's disease (AD), frontotemporal dementia, Parkinson's disease (PD), and Lewy body dementia, represent a tremendous unmet clinical need. A common feature of these diseases is the abnormal accumulation and spreading of pathological protein aggregates that play a central role, selectively affecting vulnerable circuits in a disease-specific topographic pattern. Advances in molecular imaging, such as positron emission tomography (PET) and magnetic resonance imaging (MRI), provide valuable tools for early and differential diagnosis as well as in clinical trials for screening and monitoring treatment effects. Multiplex molecular, structural and functional imaging readouts provide important etiological insights. Animal models have played important roles in the development of PET radiotracers for imaging diverse targets in AD and have enabled understanding of disease mechanisms. Research efforts have been made to map the molecular alterations and spreading of proteinopathies as well as the links with functional and structural changes as proxies for neurodegeneration.

## Molecular imaging

Chen et al. summarized the diverse AD animal models (from rodents to large animals such as non-human primates) used in preclinical imaging studies and critically assessed the pros and cons of these animal models in reflecting human disease pathophysiology. Chen et al. further provided a comprehensive current state-of-the-art review on preclinical PET imaging studies in animal models of AD, with a focus on amyloid-beta (Aβ), tau, and glucose metabolism using synaptic vesicle glycoprotein 2A (SV2A), microgliosis, neurotransmitter alterations, including N-methyl-D-aspartate (NMDA) glutamate receptors and the cholinergic system.  Maschio and Ni. reviewed recent developments in PET tracers targeting Aβ and tau in patients with AD and primary tauopathies, with a focus on the *in vitro* discovery of binding properties, off-target binding, and characteristics of different tracers.

Kong et al. summarized the potential involvement of SV2A in AD pathogenesis and the recent developments in PET tracers for SV2A as a unique imaging biomarker for synaptic density. SV2A is involved in several critical events, such as the production of Aβ species, tau hyperphosphorylation, and synaptic transmission through the calcium-related pathway in AD pathogenesis. Imaging of SV2A enables non-invasive detection of synaptic density and therefore has provided clinical evidence for synaptic loss in living AD patients.

Harada et al. summarized the recent development of PET tracers for reactive astrogliosis. Astrogliosis is a common pathology observed in brains with neurodegenerative disorders. *In vivo* imaging of reactive astrogliosis is potentially useful for early and differential diagnosis, assessment of disease severity, and evaluation of drug efficacy. Monoamine oxidase-B (MAO-B) and imidazoline_2_ binding site (I_2_BS) are two main targets for astrogliosis imaging. The authors have highlighted the development of PET tracer and that [^18^F]SMBT-1 is currently the best imaging tracer for astrogliosis for its high target-selectivity and brain permeability, low non-specific binding, and favorable reversible kinetics.

The accumulation of misfolded α-synuclein precedes the loss of dopaminergic neurons in the substantia nigra and thus is an important target for early diagnosis of PD. There is currently an unmet clinical need for biomarkers capable of imaging α-synuclein inclusions with high sensitivity and specificity. Uzuegbunam et al. developed novel [^18^F] labeled diarylbisthiazole (DABTA)-based radiotracers for detecting α-synuclein fibrils. The *in vitro* binding properties of lead tracers collaborated with the *in silico* and machine learning-predicted values. The authors further reported *in vivo* brain uptake of the tracers in wild-type mice. These findings warrant further investigation of the binding pattern of the lead tracers in postmortem brain tissues from patients with various neurodegenerative diseases, and in animal models of α-synucleinopathy.

## Functional imaging

Hugon et al. demonstrated the impact of donepezil, a widely used acetylcholinesterase inhibitor in patients with AD, on the cerebral [^18^F]fluoroglucose (FDG) uptake in a mouse model with intracerebroventricular injection of Aβ peptide. The authors established a PET signal acquisition method for [^18^F]FDG in awake/anesthetized animals and found that donepezil restored the decrease in cerebral glucose metabolism induced by pre-aggregated Aβ. This preclinical experiment supported the clinical findings of decreased cerebral glucose metabolism in patients with AD and further enabled non-invasive evaluation of the effect of medical intervention on energy metabolism (Hugon et al.).

## Structural imaging

Regional atrophy in the cortex and hippocampus has been reported earlier in various tau mouse models. Using high-field structural MRI, Sartoretti et al. found cervical spinal cord atrophy in the female transgenic pR5 mouse model of tauopathy (P301 L mutation, Thy1.2 promoter) compared to non-transgenic littermates at the age of 8.5–9 months. The author further noted a sex and transgene-dosing effects (homozygotes vs. hemizygotes) on the degree of volumetric reduction in this model (Sartoretti et al.).

Using quantitative MRI approaches, Klietz et al. demonstrated cerebral microstructural alterations in patients with early PD compared to healthy controls, including decreased regional relative proton density, and increased regional T_1_ and T_2_ relaxation time. The distinct microstructural changes detected by MRI may relate to clinical symptoms and have the potential to assist the diagnosis of PD. Further large-scale, and follow-up studies using automated volumetric analysis may further establish the utility of the proposed method (Klietz et al.).

## Author contributions

All authors listed have made a substantial, direct, and intellectual contribution to the work and approved it for publication.

## Conflict of interest

The authors declare that the research was conducted in the absence of any commercial or financial relationships that could be construed as a potential conflict of interest.

## Publisher's note

All claims expressed in this article are solely those of the authors and do not necessarily represent those of their affiliated organizations, or those of the publisher, the editors and the reviewers. Any product that may be evaluated in this article, or claim that may be made by its manufacturer, is not guaranteed or endorsed by the publisher.

